# Supplementary material for: Health care utilization and the associated costs attributable to cardiovascular disease in Ireland: a cross-sectional study
Source: Eur Heart J Qual Care Clin Outcomes. 2024 Feb 21;11(1):37–46. doi: 10.1093/ehjqcco/qcae014 (PMC11736149; doi:10.1093/ehjqcco/qcae014)
Supplement: qcae014_Supplemental_Files [file qcae014_supplemental_files.zip › Additional file 1.pdf]

Model	Ordinary Least Squares	Poisson	Zero Inflated Poisson	Negative Binomial	Zero Inflated Negative Binomial
General Practitioner visits	46370.33	51318.02	48866.65	40045.3	40047.58
Accident & Emergency Department visits	16945.26	10405.19	9454.011	9207.58	9209.581
Outpatient Department visits	36021.88	32725.25	25885.44	23549.9	23551.56
Hospital overnight admissions	15872.74	9473.855	8504.034	8299.041	9477.854
