# Supplementary figures and images for: Health care utilization and the associated costs attributable to cardiovascular disease in Ireland: a cross-sectional study
Source: Eur Heart J Qual Care Clin Outcomes. 2024 Feb 21;11(1):37–46. doi: 10.1093/ehjqcco/qcae014 (PMC11736149; doi:10.1093/ehjqcco/qcae014)

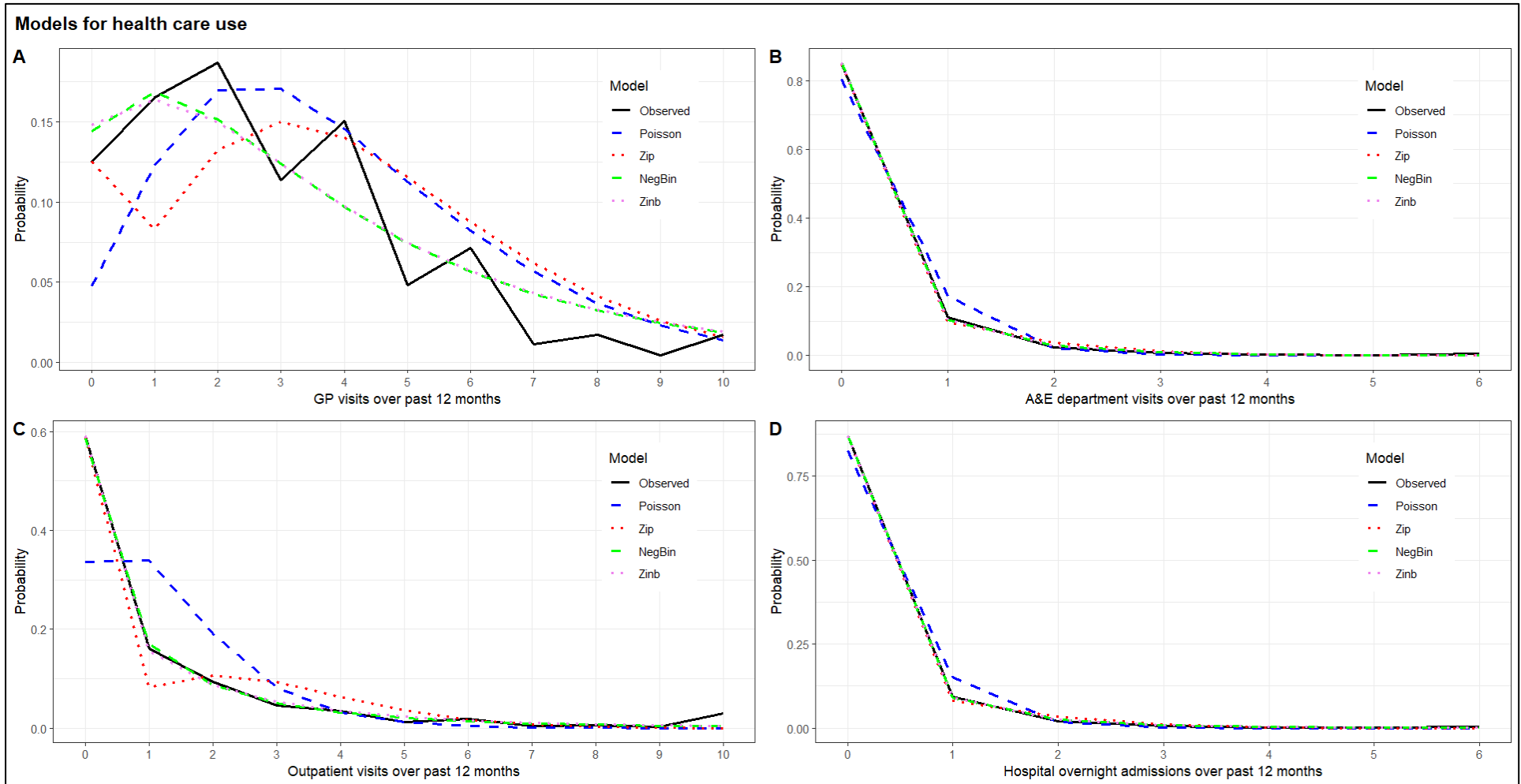

Supplement: qcae014_Supplemental_Files [file qcae014_supplemental_files.zip › Additional file 2.pdf]
